# Avian influenza surveillance reveals presence of low pathogenic avian influenza viruses in poultry during 2009-2011 in the West Bengal State, India

**DOI:** 10.1186/1743-422X-9-151

**Published:** 2012-08-07

**Authors:** Shailesh D Pawar, Sandeep D Kale, Amol S Rawankar, Santosh S Koratkar, Chandrashekhar G Raut, Satish A Pande, Jayati Mullick, Akhilesh C Mishra

**Affiliations:** 1 National Institute of Virology, Microbial Containment Complex, 130/1, Sus Road, Pashan, Pune, 411021, India; 2 Ela Foundation, C-9, Bhosale Park Sahakarnagar, Pune, 411 009, India; 3 National Institute of Virology, 20-A, Dr. Ambedkar Road, Pune, 411001, India

**Keywords:** Avian influenza surveillance, H9N2 virus, H4N6 virus, NDV, Poultry, India

## Abstract

**Introduction:**

More than 70 outbreaks of the highly pathogenic avian influenza (HPAI) H5N1 have been reported in poultry in the western and north-eastern parts of India. Therefore, in view of the recent HPAI H5N1 outbreaks in poultry, active AI surveillance encompassing wild, resident, migratory birds and poultry was undertaken during 2009–2011 in the State of West Bengal.

**Methods:**

A total of 5722 samples were collected from West Bengal; 3522 samples (2906 fecal droppings + 616 other environmental samples) were from migratory birds and 2200 samples [1604 tracheal, cloacal swabs, environmental samples, tissue samples + 596 blood (serum)] were from domestic ducks and poultry. All tracheal, cloacal and environmental samples were processed for virus isolation. Virus isolates were detected using hemagglutination assay and identified using hemagglutination inhibition (HI) and reverse transcriptase polymerase chain reaction (RT-PCR) assays. Sequencing and phylogenetic analysis of partial region of the hemagglutinin and neuraminidase genes was done. Intravenous pathogenicity index assays were performed in chickens to assess pathogenicity of AI virus isolates. Serum samples were tested for detection of antibodies against AI viruses using HI assay.

**Results:**

A total of 57 AI H9N2, 15 AI H4N6 and 15 Newcastle Disease (NDV) viruses were isolated from chickens, from both backyard and wet poultry markets; AI H4N6 viruses were isolated from backyard chickens and domestic ducks. Characterization of AI H9N2 and H4N6 viruses revealed that they were of low pathogenicity. Domestic ducks were positive for antibodies against H5 and H7 viruses while chickens were positive for presence of antibodies against AI H9N2 and NDV.

**Conclusions:**

In the current scenario of HPAI H5N1 outbreaks in West Bengal, this report shows presence of low pathogenic AI H9N2 and H4N6 viruses in chickens and domestic ducks during the period 2009–2011. This is the first report of isolation of H4N6 from India. Antibodies against AI H5 and H7 in ducks highlight the probable role of domestic ducks in the transmission of AI viruses. Human infections of H9N2 have been reported from China and Hong Kong. This necessitates implementation of prevention and control measures to limit the spread of AI viruses.

## Introduction

Avian Influenza (AI) surveillance in domestic and wild bird populations is critical to our understanding of the persistence, transmission and evolution of these viruses [1]. Type A influenza viruses belong to the family Orthomyxoviridae. They are divided into subtypes based on the serogroupings of 16 hemagglutinin and 9 neuraminidase genes. Wild aquatic birds, such as geese, shorebirds and wild ducks are the natural reservoirs of influenza A viruses [2]. AI viruses are broadly classified as low pathogenic AI (LPAI) and highly pathogenic AI (HPAI) viruses, based on their pathogenicity [3].

India reported outbreaks of the highly pathogenic avian influenza (HPAI) H5N1 in poultry in the states of Maharashtra, Gujarat, Madhya Pradesh in the western region and in Manipur, West Bengal, Tripura and Assam in the Eastern and North Eastern region during the period spanning from 2006 to 2011 [4,5]. In India HPAI H5N1 viruses were first detected in poultry in Maharashtra state in the year 2006 [6,7]. This virus re-emerged in the West Bengal state in 2008 and H5N1 outbreaks have been reported from this region. The state of West Bengal (latitude 23° 00' N and longitude – 87° 00' E) has landmass of 88,752 square kilometres (34,267 sq mi) and is known for diverse flora and fauna. Domestic ducks are known to play a major role in the spread of the H5N1 viruses where chickens and ducks often flock together and share the same water body. In India, West Bengal has the highest population of ducks along with backyard poultry [8]. Outbreaks of HPAI H5N1 have been reported from the neighboring country Bangladesh [5].

One of the best ways to intercede a possible pandemic is through systematic surveillance. Growing concerns about AI and its impact on agriculture and human health have highlighted the need to understand the role of wildlife in maintaining and spreading the virus [9]. Long-term screening and surveillance of wild, migratory birds and poultry for the presence of AI virus is imperative as a part of wider range of pandemic preparedness [10]. AI surveillance in wild birds may be useful for risk assessments in poultry, humans, pigs, and other animals [11]. Therefore, in view of the recent HPAI H5N1 outbreaks in poultry in India, active AI surveillance encompassing wild, resident, migratory birds and poultry was undertaken during 2009–2011 by the National Institute of Virology jointly with Ela Foundation and Animal Resources Development Department, Government of West Bengal. This report presents findings of the AI surveillance study.

## Materials and Methods

### Collection of samples

**Figure 1:**
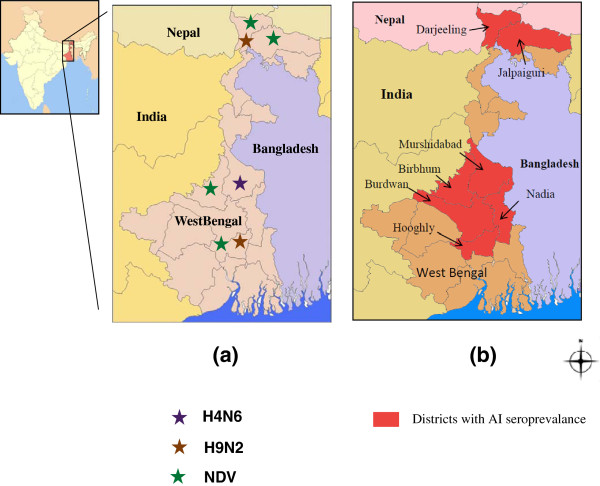
**a. AI H4N6, H9N2 and NDV isolated from various sites from districts of West Bengal b.** District-wise seropositivity against AI viruses in West Bengal.

The samples were collected from wild, resident, migratory birds and poultry during avian winter migratory season October to March in the years 2009–2011 in West Bengal (Figure 1). The screened birds belonged to 76 species from 30 avian families (Additional file 1: Table S1).

### Sample collection from wild and migratory birds

A total of 3522 samples from migratory birds were collected from ten districts and 38 locations of West Bengal including the localities around regions, which had reported HPAI H5N1 outbreaks. The samples were also collected from the wetlands near poultry farms or wetlands on the known avian migratory flyways (Table 1). The samples included fecal droppings (2906 no.) from wild, migratory birds and 616 environmental samples. Only fresh and wet samples were collected. All avian species were identified following the standard field guides. The birds were first identified and droppings were collected with sterile swabs or spoons. Aseptic precautions like wearing latex gloves, facemasks and correct disposal of used equipment were carried out [10]. As far as possible only pure flocks of wild and migratory birds were screened. When mixed flocks of migratory birds were encountered, names of all the identified species composing such flocks were entered for such samples. Samples for virus isolation were collected in viral transport medium [VTM (Hank’s balanced salt solution with Penicillin, Streptomycin, Gentamycin, Amphotericin B)], immediately sealed and transported in cold chain to the laboratory [10,12]. 

**Table 1:** Samples collected and isolation of avian influenza (AI) and Newcastle Disease Virus (NDV)

**Sr. No.**	**Locations**	**No. of samples**	**No. of isolates (%)**	
			**AI**	**NDV**
	**Wild/migratory bird samples**			
1	Ten districts*	3522	0	0
	**Samples from poultry**			
1	Murshidabad District	299	15 (5%)	0
2	Nadia District	101	0	0
3	Naxabadi (Darjeeling District)	172	0	0
	Siliguri (Darjeeling District)	152	56 (37%)	2 (1.3%)
4	Jalpaiguri District	152	0	3 (2%)
5	Hooghly District	221	0	6 (3%)
6	Kolkata (24 Paraganas)	81	1 (1.2%)	1 (1.2%)
7	Birbhum District	297	0	3 (1.0%)
8	Burdhman District	129	0	0
	**Total**	**5126**	**72 (1.4%)**	**15 (0.3%)**

* Murshidabad, Malda, Uttar Dinajpur, Dakshin Dinajpur, Birbhum, Nadia, Bardwan, Kolkata, North 24 Paraganas, South 24 Paraganas.

### Collection of samples from domestic ducks and poultry birds

A total of 1604 samples were collected from ducks and chickens from eight districts (Table 1). Cloacal, tracheal swabs and environmental samples were collected from backyard chickens and wet poultry markets and domestic ducks (*Anas platyrhynchos*). For serological analysis 596 blood (serum) samples were collected from domestic ducks (558) and chickens (38) through the wing vein. Samples for virus isolation and serology were stored at −80°C and −20°C respectively.

### Virus isolation

A total of 5126 samples (3522 samples migratory birds + 1604 samples from domestic ducks and poultry) were processed for virus isolation in 10-day-old embryonated chicken eggs (Venkateshwara Hatcheries Pvt. Limited, Pune, India). The contents of each collection vial were mixed and each vial was centrifuged at 400 g for 5 min to remove debris. Supernatant was mixed with equal quantity of viral transport medium. Each sample (200 μl) was inoculated in single egg by the allantoic route. Eggs were incubated at 37°C for 48–72 hours, were chilled at +4°C overnight, and allantoic fluids were harvested [12].

### Virus detection and identification

#### Hemagglutination (HA) and hemagglutination inhibition (HI) assays

Allantoic fluid from each egg was tested in hemagglutination assay (HA) to detect the presence of virus using 0.5% turkey and 1% horse red blood cells (RBCs). RBCs from both horse and turkey were separately used in the HA and the HI assays [12]. HI assay was performed for virus identification using influenza A H1 to H16 reference antisera (OIE/FAO National Reference Laboratory for AI and Newcastle disease, Legnaro, Italy).

### Identification of AI viruses by reverse transcriptase polymerase chain reaction (RT-PCR)

Viral RNA was extracted from the egg-isolates using the RNAeasy Viral RNA Mini kit (Qiagen, Germany). A One-Step reverse transcription-Polymerase Chain Reaction (RT-PCR) (Qiagen, Germany) was carried out for hemagglutinin and neuraminidase subtyping.

### Hemagglutinin (HA) subtyping

As these isolates were identified by HI as H9 and H4, the HA gene of these isolates were amplified using the type A-specific primers [12]. The partially amplified HA gene was sequenced (359 base pairs for H4 and 673 base pairs for H9) and compared with sequences deposited in databases. Accession numbers of H9 are JX310065 to JX310067 and H4 viruses are JX310059, JX310061 and JX310062.

### Neuraminidase (NA) subtyping

Samples which were positive for H9 and H4 were amplified using the NA diagnostic (N1 to N9) primers and run on the gel to identify the NA subtype [13]. Sequence of the NA PCR fragments (278 base pairs for N2 and 264 base pairs for N6) were compared with the available NA sequences in databases. Accession numbers of H9 are JX310068 to JX310070 and H4 viruses are JX310060, JX310063 and JX310064.

### Serology

Out of 596 (558 from domestic ducks + 38 from chickens) serum samples, 590 samples were treated with receptor destroying enzyme (Denka Seiken, Japan) for removal of non-specific inhibitors. The quantity of six serum samples was not adequate for the assay. In the serum control sera without non-specific agglutinins showed button formation, whereas sera with non-specific agglutinins showed hemagglutination. Serum samples showing hemagglutination were adsorbed with horse and turkey RBCs separately. One volume of packed RBCs were mixed with 20 volume of RDE treated serum and incubated at 4°C for 1 hour, centrifuged at 200 g for 10 minutes. Adsorbed serum was carefully removed without disturbing packed cells and used in the HI assay. The final dilution of the serum was 1:10. Titers were reported as the reciprocal of the highest dilution showing complete inhibition. Two-fold dilutions of sera were made starting with 1:10 and the highest dilution of 1:1280. A/Ck/India/NIV/2006 (H5N1), A/Ck/Italy/1067/V99 (H7N1), A/Ck/Pune/India/099321/2009 (H9N2) and Newcastle Disease virus (NDV) antigens were used for detection of antibodies against AI viruses. Results were calculated with antibody titer cut-offs 10, 20, 40 and 80. HI antibody titer of ≥20 or more was considered as seropositive for AI viruses. For NDV antibody titer of ≥10 or more was considered as seropositive [14].

### Pathogenicity studies in chickens

Representative isolates of AI H9N2 and H4N6 namely A/Ck/WB/India/1057183/2010 (H9N2) and A/Dk/WB/India/10736/2010 (H4N6) were screened for pathotyping by intravenous pathogenicity index (IVPI) assay in chickens. IVPI assay was performed as per the standard protocols [12]. AI H9N2 and H4N6 viruses showed HA titers 1024 and 64 HAU, respectively. AI H9N2 and H4N6 were also titrated in embryonated chicken eggs and showed 10^8.5^ and 10^7^ 50% egg infectious dose (EID_50_) titers. The Institutional Animal Ethical Committee approved the animal experiments. Briefly, the egg-grown isolates of AI H9N2 and H4N6 were diluted 1:10 with sterile PBS (pH 7.2) and were inoculated intravenously into ten 4–6 weeks old chickens. Two control chickens were inoculated only with PBS. All birds were kept in isolators maintained under negative pressure and observed for 10 days. The IVPI was calculated based on the scoring on symptoms/death of infected birds. At the end of observation period, cloacal swabs were collected for virus re-isolation and blood (serum) for antibody detection.

## Results

### Virus isolation and identification

None of the samples collected from wild and migratory birds were positive for AI or any other virus. Out of 1604 poultry samples, 87 samples were positive by HA test using 0.5% turkey RBCs. A total of 57 virus isolates were serologically identified as influenza AI (H9), 15 isolates as AI (H4) and 15 isolates as NDV (Table 1). These isolates showed ≥160, ≥40 and ≥640 titers in HI virus identification assay with the reference anti-sera AI (H9), AI (H4) and NDV respectively. The identified virus isolates did not show any titer with other AI reference antisera.

**Figure 2:**
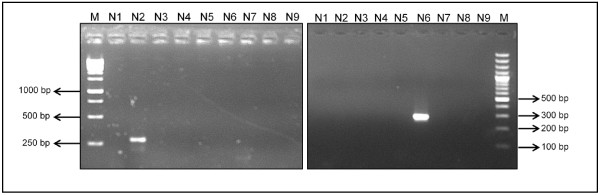
Molecular subtyping of AI H9 and H4 viruses by RT-PCR with NA subtyping primers (N1 to N9).

RT-PCR of the RNA extracted from the egg-isolates of the H9 viruses showed a distinct band with the N2 subtype while the H4 viruses distinctively showed the N6 subtype (Figure 2). Further, partial sequencing of the HA and NA genes of H9 and H4 isolates confirmed these isolates as AI H9N2 and H4N6 subtypes.

### Pathogenicity studies in chickens

In the IVPI assay, AI H9N2 and H4N6 viruses did not show signs of sickness or respiratory illness in the inoculated chickens during the observation period of 10 days. The IVP indices for all the isolates were 0.0/3.0 and hence were LPAI. Both AI H9N2 and H4N6 viruses could be re-isolated from cloacal swabs of infected birds. Antibodies were also detected in the infected birds (HI antibody titers against H9N2: >640; H4N6: >40). Virus re-isolation and the presence of antibodies against inoculated viruses confirmed infection of these birds by the inoculated viruses.

### Serology

**Figure 3:**
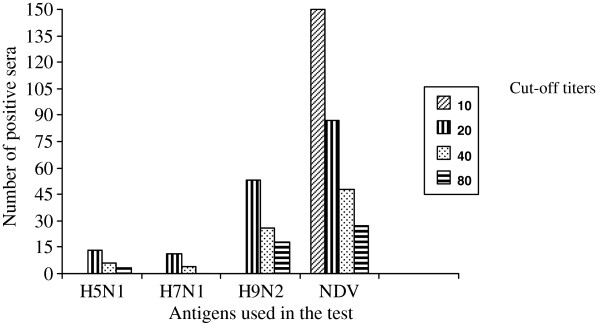
Seropositivity against AI viruses in duck and chicken sera using hemagglutination inhibition (HI) assay with different cut off titers.

A total of 227 serum samples were positive for the presence of antibodies against either HPAI A(H5N1), A(H7N1), A(H9N2) or NDV. The percent positivity for AI (H5N1), (H7N1), (H9N2) and NDV was 2.2%, 1.9%, 9.0% and 25.4%, respectively (Figure 3). Chickens were negative for antibodies against AI H5 subtype, but were positive for antibodies against NDV. The detection of ND antibodies in chickens could be due to vaccination.

## Discussion

The repeated introductions of H5N1 virus into India since 2006 have indicated the need for monitoring the wild, migratory birds and poultry population. East and Central Asian flyways of migratory birds, which include India in their path, overlap extensively in West China (around Qinghai Lake), Mongolia and Central Siberia allowing possible interchange of diseases between these areas and particularly with India [15,16]. Ringing data confirms that migratory birds like Bar-headed Geese and other species migrate from Mongolia to India during winter [15,17]. There are few reports of AI surveillance from India. The isolation and characterization of AI A(H11N1) virus from a wild aquatic bird, Eurasian Spoonbill (*Platalea leucorodia*) has been reported from India [10,18]. Though it is believed that migratory birds may have a role to play in the introduction of different AI viruses in the country, there is no data from wild, migratory birds and in ducks in West Bengal with regard to the AI viruses harboured in them. During the present study, fecal and environmental samples were collected from 76 species from 30 families of wild and migratory birds. These samples were collected during the avian winter migratory season, during which migratory birds visit India. However, no AI viruses were isolated from any of the wild bird samples. In poultry, in contrast, the present study showed that low pathogenic AI (H9N2) was the predominant AI strain circulating in the wet poultry markets and in backyard poultry in West Bengal. The partial sequencing and phylogenetic analysis of HA and NA genes of AI H9N2 showed that H9N2 viruses belonged to G1 lineage and were similar to H9N2 viruses from Iran, Saudi Arabia and Pakistan (data not shown). H9N2 viruses have been reported from India [19] and Asian countries including Bangladesh, Iran, and Pakistan have reported this virus. The seroprevalence of AI H9N2 and NDV have been reported in emus (*Dromaius novaehollandiae*) from India [20].

This is the first report of isolation of AI H4N6 virus from India. The partial sequencing and phylogenetic analysis of HA and NA genes of AI H4N6 showed that these viruses were similar to Eurasian lineage viruses (data not shown). Low pathogenic H4N6 viruses have been isolated from live bird markets in Thailand [21]. The AIV subtype H4N6 is one of the most common subtypes found through surveillance of wild waterfowl in North America [11]. The interspecies transmission of an avian H4 influenza virus to domestic pigs under natural conditions has been reported in Canada [22]. Antibodies against H4N6 have been demonstrated in wild-caught raccoon in the USA [23].

NDV infection is considered as one of the two most important diseases of chickens along with highly pathogenic avian influenza. It is an economically important disease causing heavy production loss to the farmers besides high mortality. Depending upon the pathotype and susceptibility of birds the mortality varies from zero to 100%. The disease in India is present in endemic form with frequent outbreaks in commercial poultry. Besides commercial poultry, the disease also affects the backyard poultry and it remains as a constant threat [24]. A very high seroprevalence (83%) of NDV has been reported from India [25]. NDV vaccination is routinely practiced in poultry. The present study was performed for avian influenza, during which NDV viruses were also isolated, as they grow in embryonated chicken eggs. As the priority was to characterize AI viruses, NDV were not further characterized. Further characterization of the NDV viruses isolated from this study would throw more light about their pathotyping.

Ducks showed antibodies against the H5, H7 and H9 subtypes, indicating their exposure to these AI viruses in the past. This is the first report showing the presence of antibodies against AI H5 and H7 subtypes in West Bengal. However the number of chicken sera was few as the primary objective of this study was screening of domestic ducks. Further studies with more number of chicken sera are required.

The present study does not report any HPAI H5N1 virus from the sampled birds during the study period. There is only one report of isolation of HPAI H5N1 virus from jungle crow in Assam [26]. The place from which the dead crows were recovered was 8 km from the epicentre of H5N1 outbreak in poultry. Probably these wild resident birds picked up the virus from the poultry and travelled for a short distance and died. India recently reported outbreaks of HPAI H5N1 virus in crows in Jharkhand state in November 2011 [27].

Because of legal restrictions, logistics and difficulties in trapping of the wild and migratory birds, larger sample sizes of cloacal, tracheal swabs and blood samples are difficult to obtain. Therefore, during this AI surveillance mostly environmental samples were collected and screened. It has been reported that AI H5N1 virus can replicate in feather epidermal cells in asymptomatic domestic ducks and larger amounts of viruses can be isolated for a longer time from feathers than from swabs [28]. Therefore use of feathers for diagnostic examination of HPAI H5N1 viruses during surveillance could be considered.

Reassortment between influenza H9N2 and H5N1 in poultry have been reported [29]. The high prevalence of AI H9N2 in poultry market may provide the opportunity of human infections and the possibility of reassortment with the existing poultry AI viruses including HPAI H5N1, which cannot be ruled out. In immunosuppressed chickens the H9N2 virus causes severe respiratory tract infections with high mortality in young chicks and severe decline in egg production in laying chickens, which results in economic loss. The H9N2-infected birds also shed virus to non-affected flocks through fecal-oral route without showing much of severe clinical signs [30].

Occupational exposure to infected poultry has been an important factor in AI virus transmission to humans. Human infections with H9N2 and antibodies against H9N2 have been reported in Hong Kong, China, and India [31-33]. Therefore, surveillance in poultry workers and cullers is required to trace AI virus infections from poultry. Regular cleaning and disinfection of wet poultry markets have been found to be helpful in preventing chain of transmission of AI viruses in Indonesia [34]. Such attempts would also help to curtail the spread of AI viruses in wet poultry markets. This study underlines the need of continuous active surveillance in wild, migratory birds and in poultry.

## Conclusions

This study shows presence of low pathogenic AI H9N2 and H4N6 viruses in chickens and domestic ducks during the period 2009–2011 in the state of West Bengal, India. Antibodies against AI H5 and H7 in ducks highlight the probable role of domestic ducks in the transmission of AI viruses. In the current scenario of emerging influenza viruses, continuous monitoring and characterization of AI viruses in wild, migratory, resident birds and poultry is required to limit spread of AI viruses.

## Competing interests

The authors declare that they have no competing interests.

## Authors’ contribution

SDP, SAP and ACM conceived and designed the field, experimental work. SDP, SDK, ASR, SSK, CGR, SAP and JM performed the field and experimental work. SDP, SDK, ASR, SSK, CGR, SAP, JM and ACM analyzed the data. SDP, JM and ACM wrote the paper. All authors read and approved the final manuscript.

## Supplementary Material

Additional file 1 **Table S1.** Wild avian species sampled during avian influenza surveillance in West Bengal. Click here for file
