# Supplementary material for: Avian influenza surveillance reveals presence of low pathogenic avian influenza viruses in poultry during 2009-2011 in the West Bengal State, India
Source: Virol J. 2012 Aug 7;9:151. doi: 10.1186/1743-422X-9-151 (PMC3488339; doi:10.1186/1743-422X-9-151)
Supplement: Additional file 1 — Table S1. Wild avian species sampled during avian influenza surveillance in West Bengal. [file 1743-422X-9-151-S1.doc]

**Supplementary Table:** Wild avian species sampled during avian influenza surveillance in West Bengal

| **Common Name** | **Scientific Name** | **Family** | **Status** |
| --- | --- | --- | --- |
| Little Grebe | *Tachybaptus ruficollis* | Podicipedidae | R |
| Little Cormorant | *Phalacrocorax niger* | Phalacrocoracidae | R |
| Great Cormorant | *Phalacrocorax carbo* | Phalcrocoracidae | R |
| Indian Shag | *Phalacrocorax fuscicollis* | Phalcrocoracidae | R |
| Pond Heron | *Ardeola grayii* | Ardeidae | R |
| Little Green Heron | *Butorides striatus* | Ardeidae | R |
| Yellow Bittern | *Ixobrychus sinensis* | Ardeidae | R |
| Cattle Egret | *Bubulcus ibis* | Ardeidae | R |
| Little Egret | *Egretta garzetta* | [Ardeidae](http://en.wikipedia.org/wiki/Ardeidae) | R |
| Median Egret | *Mesophoyx intermedia* | Ardeidae | R |
| Grey Heron | *Ardea cinerea* | [Ardeidae](http://en.wikipedia.org/wiki/Ardeidae) | R/M |
| Purple Heron | *Ardea purpurea* | Ardeidae | R |
| Asian Openbill Stork | *Anastomus oscitans* | Ciconiidae | R/M |
| White-necked Stork | *Ciconia episcopus* | Ciconiidae | R |
| Lesser Adjutant-Stork | *Leptoptilos javanicus* | Ciconiidae | R |
| Greylag Goose | *Anser anser* | Anatidae | M |
| Lesser Whistling Duck | *Dendrocygna javanica* | Anatidae | R |
| Cotton Teal | *Nettapus coromandelianus* | Anatidae | R/M |
| Northern Pintail | *Anas acuta* | Anatidae | M |
| Tufted Pochard | *Aythya fuligula* | Anatidae | M |
| Garganey | *Anas querquedula* | Anatidae | M |
| Eurasian Wigeon | *Anas penelope* | Anatidae | M |
| Black-shouldered Kite | *Elanus caeruleus* | Accipitridae | R |
| Western Marsh Harrier | *Circus aeruginosus* | Accipitridae | M |
| Common Moorhen | *Gallinula chloropus* | Rallidae | R |
| White-breasted Waterhen | *Amaurornis phoenicurus* | Rallidae | R |
| Purple Moorhen | *Porphyrio porphyrio* | Rallidae | R |
| Common Moorhen | *Gallinula chloropus* | Rallidae | R |
| Common Coot | *Fulica atra* | Rallidae | R |
| Bronze-winged Jacana | *Metopidius indicus* | Jacanidae | R |
| Pheasant-tail Jacana | *Hydrophasianus chirurgus* | [Jacanidae](http://en.wikipedia.org/wiki/Jacanidae) | R |
| Pacific Golden Plover | *Pluvialis fulva* | Charadriidae | M |
| Lesser Sand Plover | *Charadrius mongolus* | Charadriidae | M |
| Grey-headed lapwing | *Vanellus cinereus* | Charadriidae | M |
| Red-wattled Lapwing | *Vanellus indicus* | Charadriidae | R |
| Northern Lapwing | *Vanellus vanellus* | Charadriidae | M |
| Common Sandpiper | *Actitis hypoleucos* | [Scolopacidae](http://en.wikipedia.org/wiki/Scolopacidae) | M |
| Green Sandpiper | *Tringa ochropus* | [Scolopacidae](http://en.wikipedia.org/wiki/Scolopacidae) | M |
| Wood Sandpiper | *Tringa glareola* | [Scolopacidae](http://en.wikipedia.org/wiki/Scolopacidae) | M |
| Marsh Sandpiper | *Tringa stagnatilis* | [Scolopacidae](http://en.wikipedia.org/wiki/Scolopacidae) | M |
| Jack Snipe | *Lymnocryptes minimus* | Scolopacidae | M |
| Common Redshank | *Tringa totanus* | Scolopacidae | M |
| Whimbrel | *Numenius phaeopus* | Scolopacidae | M |
| Common Greenshank | *Tringa nebularia* | Scolopacidae | M |
| Ruddy Turnstone | *Arenaria interpres* | Scolopacidae | M |
| Black-winged Stilt | *Himantopus himantopus* | Recurvirostridae | M |
| Brown-headed Gull | *Larus brunnicephalus* | Laridae | M |
| Black-headed Gull | *Larus ridibundus* | Laridae | M |
| Heuglin’s Gull | *Larus heuglini* | Laridae | M |
| Little Tern | *Sterna albifrons* | Sternidae | R/M |
| Spotted Dove | *Streptopelia chinensis* | Columbidae | R |
| Rose-ringed parakeet | *Psittacula krameri* | Psittacidae | R |
| Greater Coucal | *Centropus sinensis* | Cuculidae | R |
| White-throated Kingfisher | *Halcyon smyrnensis* | Alcedinidae | R |
| Small-blue Kingfisher | *Alcedo atthis* | Alcedinidae | R |
| Lesser Pied Kingfisher | *Ceryle rudis* | Cerylidae | R |
| Indian Roller | *Coracias benghalensis* | Coraciidae | R |
| Hoopoe | *Upupa epops* | Upupidae | R |
| Blue-throated Barbet | *Megalaima asiatica* | Capitonidae | R |
| Barn Swallow | *Hirundo rustica* | Hirundinidae | M |
| Paddyfield Pipit | *Anthus rufulus* | Motacillidae | R |
| Pied Wagtail | *Motacilla alba* | Motacillidae | M |
| Large Pied Wagtail | *Motacilla maderaspatensis* | Motacillidae | R |
| Red-vented Bulbul | *Pycnonotus cafer* | Pycnonotidae | R |
| Long-tailed Shrike | *Lanius schach* | Laniidae | R |
| Black Redstart | *Phoenicurus ochruros* | Muscicapidae | M |
| Jungle Babbler | *Turdoides striatus* | Timaliidae | R |
| Pied Starling | *Gracupica contra* | Sturnidae | R |
| Common Myna | *Acridotheres tristis* | Sturnidae | R |
| Jungle Myna | *Acridotheres fuscus* | Sturnidae | R |
| Bank Myna | *Acridotheres ginginianus* | Sturnidae | R |
| Asian Pied Starling | *Sturnia contra* | Sturnidae | R |
| Black Drongo | *Dicrurus macrocercus* | Dicruridae | R |
| Rufous Treepie | *Dendrocitta vagabunda* | Corvidae | R |
| Jungle Crow | *Corvus culminatus* | Corvidae | R |
| House Crow | *Corvus splendens* | Corvidae | R |

**Key:** R-Resident, M-Migratory.
